# Clinical trials with anti-angiogenic agents in hematological malignancies

**DOI:** 10.1186/2040-2384-2-10

**Published:** 2010-06-22

**Authors:** Michael Medinger, Klaus Mross

**Affiliations:** 1 Department of Hematology, University Hospital Basel, Petersgraben 4, 4031 Basel, Switzerland; 2 Department of Medical Oncology, Tumor Biology Center at the Albert-Ludwigs-University, Breisacherstrasse 117, D-79106 Freiburg, Germany

## Abstract

New blood vessel formation (angiogenesis) is not only essential for the growth of solid tumors but there is also emerging evidence that progression of hematological malignancies like multiple myeloma, acute leukemias, and myeloproliferative neoplasms, also depends on new blood vessel formation. Anti-angiogenic strategies have become an important therapeutic modality for solid tumors. Several anti-angiogenic agents targeting angiogenesis-related pathways like monoclonal antibodies, receptor tyrosine kinase inhibitors, immunomodulatory drugs, and proteasome inhibitors have been entered clinical trials or have been already approved for the treatment of hematological malignancies as well and in some instances these pathways have emerged as promising therapeutic targets. This review summarizes recent advances in the basic understanding of the role of angiogenesis in hematological malignancies and clinical trials with novel therapeutic approaches targeting angiogenesis.

## Introduction

The hypothesis of tumor angiogenesis in malignancies was raised by Judah Folkman: To grow over a certain size of a few millimetres in diameter solid tumors need blood supply from surrounding vessel [1]. Up to 2-3 mm^3 ^solid tumors can grow without blood vessel supply. Nutrition and oxygen is provided via diffusion from the surrounding tissue. Above this size, diffusion becomes insufficient due to the negative surface/volume ratio. Based on a balance between angiogenic and anti-angiogenic growth factors, a tumor of this size can stay dormant for a very long time period until the so-called angiogenic switch occurs [2]. Tumor blood vessels are generated by various mechanisms, such as expansion of the host vascular network by budding of endothelial sprouts (sprouting angiogenesis), cooption of the existing vascular network, remodeling and expansion of vessels by the insertion of interstitial tissue columns into the lumen of preexisting vessels (intussusceptive angiogenesis) and homing of endothelial cell precursors (EPC; CEP) from the bone marrow or peripheral blood into the endothelial lining of neovessels (vasculogenesis) [3].

Tight control of angiogenesis is maintained by a balance of endogenous anti-angiogenic and pro-angiogenic factors [4]. VEGF has a key, rate-limiting role in promoting tumor angiogenesis and exerts its effects by binding to one of three tyrosine kinase receptors: VEGF receptor-1 (VEGFR-1; fms-like tyrosine kinase-1, Flt-1), VEGFR-2 (human kinase domain region, KDR/murine fetal liver kinase-1, Flk-1) and VEGFR-3 (Flt-4). VEGFR-1 (ligands include VEGF-A, -B and placental growth factor [PIGF]) and VEGFR-2 (ligands include VEGF-A, -C and -D) are predominantly expressed on vascular endothelial cells, and activation of VEGFR-2 appears to be both, necessary and sufficient, to mediate VEGF-dependent angiogenesis and induction of vascular permeability [4,5]. Both receptor tyrosine kinases are expressed in all adult endothelial cells, except for the brain endothelial cells. VEGFR-1 is also expressed on hematopoietic stem cells, vascular smooth muscle cells, monocytes, and leukemic cells [6,7], while VEGFR-2 is expressed on endothelial progenitor cells and megakaryocytes [8,9]. VEGFR-3, largely restricted to lymphatic endothelial cells, binds the VEGF homologues VEGF-C and VEGF-D and may play an important role in the regulation of lymphangiogenesis. Thus, VEGF and VEGFR represent significant anti-cancer therapy targets, which elegantly bypass potential tumor-related treatment barriers [4].

A further important pathway in angiogenesis is the recently identified Delta-Notch pathway, and particularly the ligand Delta-like 4 (Dll4), was identified as a new target in tumor angiogenesis [10]. Dll4 is highly expressed by vascular endothelial cells and induced by VEGF [11]. It interacts with Notch cell surface receptors to act as a negative feedback inhibitor downstream of VEGF signaling to restrain the sprouting and branching of new blood vessels [10,12]. Inhibition of Dll4-Notch signaling induces an increase in vessel density but these blood vessels are abnormal and not perfused [13]. Therefore intratumour hypoxia is increased and leads to induction of transcription of proangiogenic genes regulated by Hypoxia inducible factor-1 (HIF-1) [10,14]. Disruption of Dll4 signaling by overexpression or inhibition of Dll4 may impair angiogenesis and blockade of Dll4-Notch signaling results in an increased density of nonfunctional vasculature and is associated with a reduction in the growth of human tumor xenografts [13,14]. Further, certain xenografts that are resistant to anti-VEGF therapy are reported to be sensitive to anti-Dll4 and combination treatment with anti-VEGF and anti-Dll4 has additive inhibitory effects on tumor growth [13-15].

This review summarizes the role of pathological angiogenesis in hematological malignancies focusing on multiple myelomas (MM), acute leukemias, and myeloproliferative neoplasms (MPN) and its therapeutic intervention with novel agents within clinical trials or already approved.

## Pathophysiology of angiogenesis in hematological malignancies

Many studies suggest a role for angiogenesis not only in the pathogenesis of solid tumors but also in hematological malignancies like acute and chronic leukemia, lymphoma, myelodysplastic syndromes, myeloproliferative neoplasms, and multiple myeloma [16-21]. We and others reported an increased microvessel density and VEGF expression in the bone marrow of patients with myeloproliferative neoplasms and lymphoma [17,20]. Thereby, the extent of angiogenesis in the bone marrow often correlated with disease burden, progonosis, and treatment outcome [22,23]. In the neoplastic bone marrow there is an imbalance of the cells, cytokines and growth factors maintaining physiological angiogenesis in the normal bone marrow. The bone marrow tumor cells upregulates several factors, including interleukin-6, granulocyte-macrophage colony-stimulating factor and VEGF, have autocrine and paracrine effects acting on multiple cell types, thereby stimulating angiogenesis and leading to increased vascularity [7,24]. The role for VEGF in hematogical malignancies has been extensively studied since its isolation from the leukemia cell line HL- 60 in 1989 [25]. Apparently, this growth factor is expressed in many other leukemic cell lines [7,26] and a subset of leukemic cells also expresses VEGFR-2 which allows VEGF to act as autocrine growth factor in leukemia [26,27]. In addition to that, isolated blast cells from leukemia patients also produce VEGF [26] and the cellular level of VEGF in acute myeloid leukemia (AML) patients has been identified as independent prognostic risk factor [28]. VEGF from leukemic blasts contributes to disease progression, either as positive regulator for proliferation and apoptosis protection for the blast itself or by activating the surrounding stroma cells with subsequent induction of bone marrow angiogenesis.

Regarding the Notch pathway, Notch signals are oncogenic in hematogical malignancies in many cellular contexts [29]. Activating Notch-mutations have been shown to be present in at least 50% of human T-cell acute lymphoblastic leukaemia (T-ALL) cases and have been proved to play a unifying role in the pathogenesis of T-ALL [30]. An important role of Notch has been proposed in cell survival in several B-cell malignancies such as Hodgkin's disease [31,32] and in two B-cell non-Hodgkin lymphoma entities, chronic lymphocytic leukaemia (CLL) [33-35] and in MM [36,37].

### Multiple myeloma

MM was the first hematological malignancy, in which increased angiogenesis rate was detected [21,38]. MM is characterized by proliferation of malignant plasma cells that accumulate in the bone marrow and often produce a monoclonal immunoglobulin. New vessel formation in the bone marrow seems to play an important role in the pathogenesis of MM [39,40]. Increased bone marrow microvessel density (MVD) in patients with MM appears to be also an important prognostic factor [41]. Malignant plasma cells can secrete various cytokines, including VEGF, basic fibroblast growth factor (bFGF), and hepatocyte growth factor (HGF), all known for their pro-angiogenic activity [42]. It has been shown that MM cells are capable of secreting VEGF in response to Interleukin-6 (IL-6) stimulation; in response to that VEGF stimulation microvascular endothelial cells and bone marrow stromal cells secrete in turn IL-6, a potent growth factor for malignant plasma cells, thus closing a paracrine loop [43]. Specifically, increased microvessel density (MVD) in the BM of MM patients has been correlated with disease progression and poor prognosis [21,23]. Moreover, VEGF also exerts direct effects on MM cell migration, proliferation, survival, and drug resistance. VEGF triggered effects in MM cells are predominantly mediated via VEGFR 1 and in endothelial cells, predominantly via VEGF R2 [44]. Rajkumar et al. showed a gradual increase of bone marrow angiogenesis along the disease spectrum from monoclonal gammopathy of undetermined significance (MGUS) to smoldering MM, newly diagnosed MM and relapsed MM [45], though the expression levels of VEGF, bFGF, and their receptors were similar among MGUS, smoldering MM, and newly diagnosed MM [46], rising the hypothesis that MVD increase in plasma cell neoplasias could be rather a function of chronology.

### Acute leukemias

The first demonstration that leukemia progression might be accompanied by an increase of bone marrow vascularization was provided by Judah Folkman's group [47]. In their studies, it was demonstrated that the bone marrow of acute lymphoblastic leukemia (ALL) patients had increased blood vessel content, compared to normal counterparts. Moreover, it was also shown that urine and peripheral blood samples from ALL patients contained elevated levels of pro-angiogenic growth factors, namely bFGF and VEGF, which correlated with the increase of bone marrow angiogenesis [48]. The existence of an "angiogenesis switch", first proposed for solid tumors [49], was therefore suggested to apply to hematological malignancies as well. "Angiogenesis switch" in leukemia is documented by increased bone marrow MVD, increased expression of HIF-1, multiple pro-angiogenic factors (VEGF, bFGF, angiopoietin-2), soluble VEGFR, and decreased expression of endogenous angiogenesis inhibitors, such as thrombospondin-1 [50,51].

In a recent study by Norén-Nyström et al. [52] MVD, analyzed on 185 bone marrow biopsies, was higher in T-ALL compared to B-ALL. In the B-ALL group, cases with t(12;21) were characterized by a low MVD, while patients with hyperdiploid leukemia showed a high MVD. Similarly, in previously untreated acute myeloid leukemia (AML), increased levels of plasma VEGF correlate with reduced survival and lower remission rates [53]. In addition to that, isolated blast cells from leukemia patients also produce VEGF and the cellular level of VEGF in AML patients has been identified as independent prognostic risk factor [28]. In a reccent study [54] dynamic contrast-enhanced magnetic resonance imaging (DCE-MRI) was used as a non-invasive technique to measure bone marrow angiogenesis in AML. DCE-MRI was performed beforte treatment and on day 7 after induction chemotherapy. Thereby, bone marrow angiogenesis with remission, rate overall and disease-free survival.

### Myeloproliferative neoplasms

The available data on angiogenesis and expression of VEGF and its receptors in the bone marrow of patients with *BCR-ABL1-*negative myeloproliferative neoplasms (MPN) suggest that MVD is increased, especially in primary myelofibrosis (PMF), and that increased angiogenesis might inversely correlate with survival [55-58]. In a recent study, we found a significantly increased MVD and VEGF expression in MPN compared to controls especially in cases with high *JAK2-V617F *mutant allele burdens [17]. The identification of an acquired somatic mutation in the *JAK2 *gene, resulting in a valine to phenylalanine substitution at position 617 (*JAK2-V617F*), has provided new insights into the pathogenesis of *BCR-ABL1-*negative MPN, being present in most patients with polycythaemia vera (PV) and in about 50% of patients with essential thrombocythemia (ET) and PMF [59,60]. In another study by Alonci et al. in patients with MPN, serum levels of VEGF and VEGFR-2 was examined. In MPN, VEGF levels were higher compared to controls, wheresas VEGFR-2 levels was reduced in ET but not in PV and PMF [61].

## Anti-angiogenic therapies in hematological malignancies

**Figure 1:**
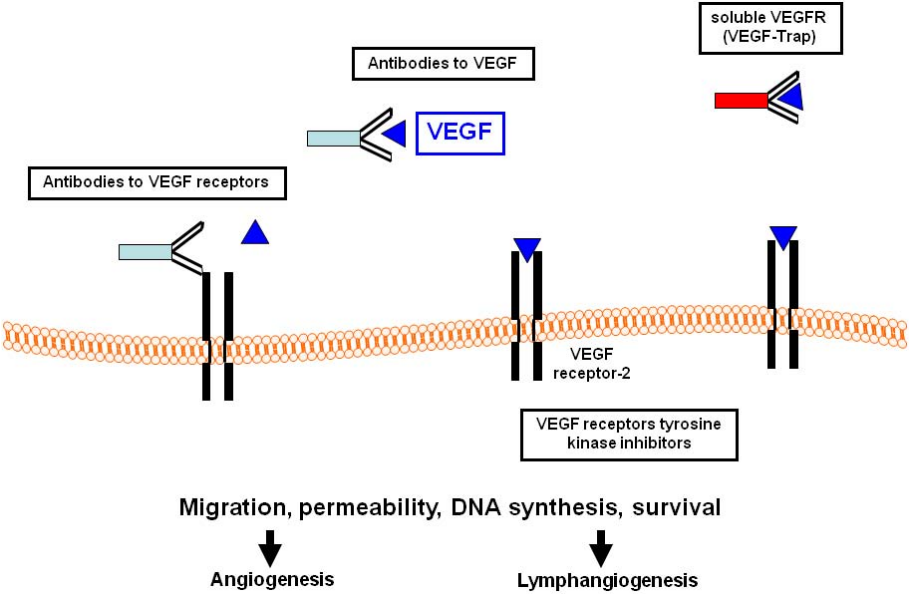
**Therapeutic strategies to target the VEGF/VEGF receptor system**. VEGF, vascular endothelial growth factor.

Anti-angiogenic therapies are mostly based on inhibiting the binding of VEGF to VEGFR by neutralizing antibodies to the ligand or to the receptor, soluble receptors, small molecule inhibitors or are directed against the tyrosine kinase activity of the VEGF receptors (Figure 1). The first anti-angiogenic agent to be approved in solid tumors was bevacizumab (Avastin™, Genentech), a humanized anti-VEGF monoclonal antibody. Administration of bevacizumab, in combination with cytotoxic chemotherapy, conferred benefits to patients with metastatic colorectal cancer, non-squamous, non-small cell lung cancer and metastatic breast cancer [62-64]. Additionally, two small-molecule inhibitors targeting VEGFRs and other kinases, sorafenib (Nexavar™, Bayer and Onyx pharmaceuticals) and sunitinib (Sutent™, Pfizer), have been approved based on their efficacy in treating renal cell- and hepatocellular carcinoma [65,66]. A growing list of anti-angiogenics is now available, either in various stages of clinical development or as components of standard clinical regimens. The major classes of anti-angiogenic therapy include: (1) direct anti-VEGF acting molecules (anti-VEGF antibodies, *VEGF*-antisense nucleotides); (2) immunomodulatory drugs (IMIDs) with antiangiogenic properties; (3) receptor tyrosine kinase inhibitors, targeting VEGFR signaling as well as receptors of other (pro-angiogenic) factors; (4) anti-endothelial approach of metronomic therapy and (5) other new compounds, targeting signaling downstream to pro-angiogenic growth factors, such as mammalian target of rapamycin (mTOR) inhibitors, histone deacetylases' (HDAC) inhibitors and proteasome inhibitors.

In our review, we will focus on several molecules interfering with the VEGF/VEGFR system, which already have been approved or are currently evaluated in clinical trials for treatment of hematological malignancies (Table 1).

**Table 1:** Selection of clinical trials and approved anti-angiogenic therapies in hematological malignancies

Drug	Target	Study entities	Approved for
**Receptor tyrosine kinase inhibitors**			

PTK787/ZK 222584 (Vatalanib^®^)	VEGFR1-3, PDGFRβ, c-Kit	AML, PMF, MDS, CML, DLBCL, MM	

SU5416 (Semaxinib)	VEGFR1-2, c-kit, Flt3	AML, MDS, MM, MPN	

Sorafenib (Nexavar^®^)	VEGFR2-3, B-Raf, Faf-1, PDGFRβ	AML, ALL, MDS, CML, CLL, NHL, MM	Advanced renal cell carcinoma, HCC

Sunitinib (Sutent^®^)	VEGFR1-3, PDGFRα+β, c-kit, Flt3	AML, MDS, CLL, Myeloma, NHL	Advanced renal cell carcinoma, GIST

PKC-412 (Midostaurin)	VEGFR2, PKC, PDGFR, Flt3, c-Kit	AML	

Cediranib (Recentin^®^)	VEGFR1-3, PDGFRβ, c-Kit	AML, MDS, CLL	

**Proteasome inhibitors**			

Bortezomib (Velcade^®^)	26S proteasome, NF-κB	AML, ALL, MDS, CML, NHL, MCL	MM, MCL

**Anti-VEGF strategies**			

Bevacizumab (Avastin^®^)	VEGF-A	AML, MDS, CLL, CML, NHL, MM	Metastatic colorectal cancer, NSCLC, breast cancer

**Immunomodulatory drugs**			

Thalidomide	bFGF, VEGF, IL-6	AML, MDS, MPN, CLL, NHL, MM	MM

Lenalidomide (Revlimid^®^)	bFGF, VEGF, IL-6	AML, MDS, CLL, NHL	MM, 5q- MDS

AML, acute myeloid leukemia; bFGF, basic fibroblast growth factor; DLBCL, diffuse large B-cell lymphoma; CLL, chronic lymphocytic leukemia; CML, chronic myeloid leukemia; GIST, gastrointestinal stromal tumors; HCC, hepatocellular carcinoma; IL-6, Interleukin-6; NHL, non-Hodgkin lymphoma; NSCLC, non-small cell lung cancer; MCL, mantle cell lymphoma; MDS, myelodysplastic syndrome; MM, multiple myeloma; MPN, myeloproliferative neoplasm; PMF, primary myelofibrosis; VEGF, vascular endothelial growth factor.

### Anti-VEGF monoclonal antibodies

#### Bevacizumab

The humanized monoclonal anti-VEGF antibody bevacizumab (Avastin^®^) is the first drug targeting VEGF and is officially approved in combination with chemotherapy. Bevacizumab is a humanized murine anti-human VEGF monoclonal IgG_1 _antibody that blocks the binding of human VEGF to its receptors, thereby disrupting also autocrine and paracrine survival mechanisms mediated by VEGFR-1 and VEGFR-2 [67]. Bevacizumab was approved for advanced non-small cell lung cancer (NSCLC), breast cancer, and colorectal cancer. In patients with refractory AML (n = 9) bevacizumab resulted in reduction of VEGF expression in the bone marrow but without a clinical response [68]. Bevacizumab-associated side effects are generally mild to moderate in severity, although there are specific, uncommon events that are more severe and potentially lifethreatening. The most commonly observed adverse events are hypertension, proteinuria, bleeding and thrombosis, which are generally mild to moderate and manageable [68]. In a phase II clinical trial by Karp et al. bevacizumab was administered after chemotherapy to adults with refractory or relapsed AML [69]. Bevacizumab 10 mg/kg was administered on day 8 after cytarabine beginning day 1 and mitoxantrone beginning day 4. Forty-eight adults received induction therapy. Overall response was 23 of 48 (48%), with complete response (CR) in 16 (33%). Eighteen patients (14 CR and 4 partial responses) underwent one consolidation cycle and 5 (3 CR and 2 partial responses) underwent allogeneic transplant. Median overall and disease-free survivals for CR patients were 16.2 months (64%, 1 year) and 7 months (35%, 1 year), respectively. As biomarkers, the microvessel density in bone marrow biopsies, serum VEGF levels and the expression of the VEGFR-1 FLT1 by AML marrow blasts were determined. The expression of FLT-1 in pretreatment AML marrow cells was higher compared to normal bone marrow. Bone marrow samples demonstrated marked MVD decrease after bevacizumab. VEGF was detected in pretreatment serum in 67% of patients tested, increased by day 8 in 52%, and decreased in 93% (67% undetectable) 2 h after bevacizumab. Currently, bevacizumab is evaluated as treatment option for newly diagnosed AML in combination with cytarabine and idarubicin in a phase II study.

#### Anticalins^©^

The anticalins represent a novel class of human binding proteins. PRS-050 is an anticalin with extended serum half-life due to pegylation. This anticalin targets VEGF and exhibits favourable binding und functional in vitro activity profile in direct comparison to the currently approved VEGF antagonists. A strong enhanced vascular permeability was demonstrated. The wordwide "first trial in man" is running since May 2010 and first results will be expected in 2011. The potential use will be similar to Bevacizumab. Its usefulness in malignant hematological disorders has to be explored after the phase I study.

#### Receptor tyrosine kinase inhibitors

Small tyrosine kinase inhibitors that target VEGFR are a further important class of anti-angiogenic drugs. Their efficacy in hematological malignancies, especially in AML, might be attributable to inhibition of a lot of pathways, especially such related to c-kit and Flt3.

SU5416 (Semaxinib) is a small molecule inhibitor of VEGFR-1 and 2, c-kit and Flt3 [70-72]. In a phase II study, 42 patients with advanced AML were treated [70]. 7 patients achieved a partial response (reduction of blasts by at least 50%), with one complete morphological response lasting 2 months. Treatment was generally well tolerated. Most study drug-related adverse events were mild to moderate in severity and the most frequently reported adverse events were nausea, bone/muscoloskeletal pain, headache, insomnia, and vomiting. As biomarkers, VEGF RNA expression by leukemic blasts, bone marrow MVD, and the expression of the target receptors c-kit, Flt3, VEGFR-1, and VEGFR-2 prior to therapy was determined. Patients with AML blasts expressing high levels of VEGF mRNA by quantitative polymerase chain reaction (PCR) had a significantly higher response rate and reduction of bone marrow MVD than patients with low VEGF expression consistent with the anti-angiogenic effects of SU5416. Patients with a high c-kit expression had a lower response.

Vatalanib (formerly PTK787/ZK 222584) is an oral protein kinase inhibitor (PTK) acting as angiogenesis inhibitor that is active against VEGFR and PDGFR tyrosine kinases, thereby offering a novel approach to inhibiting tumor growth [73]. It interferes with the ATP binding sites of VEGFR. In a phase I study by us, vatalanib was well tolerated and showed clinical activity in a variety of solid tumors [74]. It is active in MM by primarily reducing the number of tumor microvessels, accompanied by dilation of the remaining vessels [75,76]. Ongoing studies evaluate the efficacy of valatinib in combination with imatinib in a phase I/II trial for patients with AML, PMF, and blast phase of chronic myelogenous leukemia. Vatalanib was studied in a phase I clinical trial alone or in combination with cytosine-arabinoside and daunorubicin in patients with myelodysplastic syndromes (MDS) and AML [77]. Sixty-three patients received vatalanib at doses of 500-1000 mg/bid orally. At 1000 mg/bid, dose-limiting toxicities such as lethargy, hypertension, nausea, emesis and anorexia were observed. CR was observed in 5 of 17 evaluable AML patients treated with vatalanib combined with chemotherapy. The authors concluded that vatalanib is generally well tolerated and can be given in combination with chemotherapy in patients with MDS and AML. In a recently study by Barbarroja et al. [78] vatalanib was examined in combination with idarubicin in 4 AML cell lines and 7 AML patients samples. Vatalanib decreased VEGF levels and VEGFR phosphorylation in AML cells, which showed *FLT3 *internal tandem reduplications/mutations (ITD), raising the question of the actual targeted tyrosine kinase (VEGFR or flt3). In another study, vatalanib was given to 29 patients with PMF at doses of 500 or 750 mg/bid. One patient (3%) achieved CR and 5 (17%) clinical improvement. All together, vatalanib had modest activity in patients with PMF [79].

Cediranib (AZD2171, Recentin^®^) is a potent inhibitor of both VEGFR-1 and VEGFR-2; it also has activity against c-kit, PDGFR-β, and VEGFR-3 at nanomolar concentrations [80]. In our study, cediranib was well tolerated up to 45 mg/d in patients with a broad range of solid tumors [81]. The most common toxicities included diarrhea, dysphonia, and hypertension. In a phase I study with cediranib in 35 AML patients the most common adverse events were diarrhea, hypertension and fatigue. Six patients experienced an objective response (3 each at 20 and 30 mg). Dose and time-dependent reductions of soluble VEGFR-2 were observed, and there was a correlation between cediranib exposure and plasma VEGF levels [82].

#### Immunomodulatory drugs (IMiDs)

**Figure 2:**
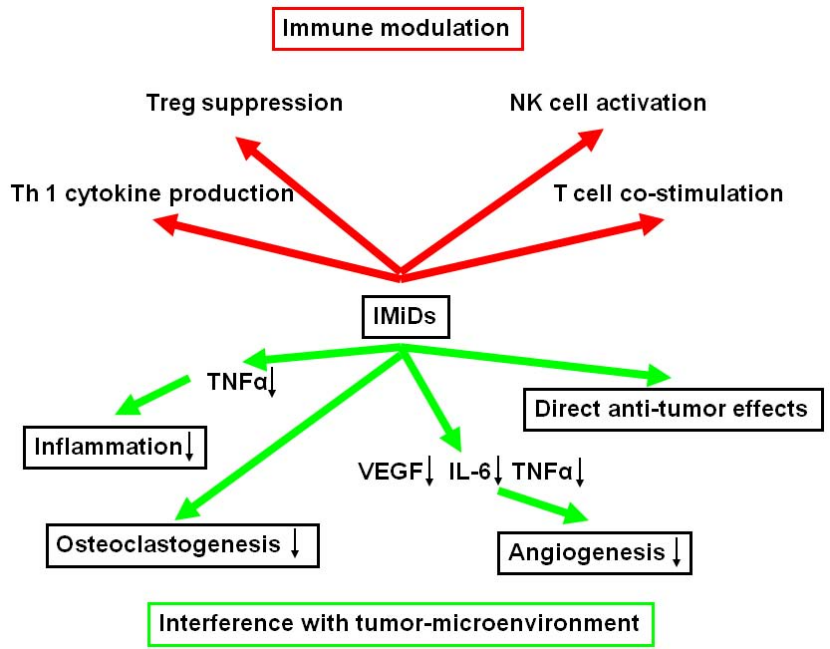
**Modes of action of IMiDs**. IL-6, interleukin-6; IMiDs, immunomodulatory drugs; NK cells, Natural killer cells; Th1 cells, T helper 1 cells; TNFα, tumor necrosis factor alpha; Treg, regulatory T cells; VEGF, vascular endothelial growth factor.

Thalidomide was originally introduced as sedative and withdrawn in the 1960's due to deleterious side effects. Recently, there is increasing evidence for the efficacy of thalidomide in cancer therapy. Multiple myeloma is one of the first clinical entities for which this could be demonstrated [83,84]. The surprising effects of thalidomide have led to the development of a series of IMiDs with even higher anti-angiogenic potency (Figure 2). It has has been shown that thalidomide has important immunomodulatory effects by decreasing TNF-α synthesis and slectively modulating T cell subsets shifting the T cell population towards T helpers [85]. The interest on thalidomide as an anti-neoplastic agent rose after demonstration of its anti-angiogenic activity in a rabbit model of corneal neovascularization that was induced in response to bFGF [86]. Thalidomide and the newer immunomodulatory drugs (IMiDs) (e.g. lenalidomide) have been shown to significantly decrease the expression of the pro-angiogenic factors VEGF and Interleukin-6 (IL-6) in MM [87]. The newer IMiDs were found to be 2-3 times more potent compared to thalidomide concerning anti-angiogenic activity in various in vivo assays [88]. The anti-angiogenic activity of IMiDs has been shown to be independent of their immunomodulatory effects [89].

Thalidomide monotherapy in a phase II trial, in which 84 patients with relapsed and refractory MM received doses ranging from 200 to 800 mg/d, resulted in an overall response rate of 32%. The 2-year event-free survival and overall survival were 20 and 48%, respectively [83,84]. In combination with dexamethasone the response rate was 63% compared to 41% with dexamethasone alone in patients with newly diagnosed MM [90]. Thalidomide was approved for the treatment of newly diagnosed MM.

In patients with AML, thalidomide was examined as mono- and combination therapy. In a phase II study by Thomas et al. [91] thalidomide was analyzed in patients with relapsed or refractory AML previously treated with cytarabine-containing regimens. A total of 16 patients were treated with oral thalidomide 200-800 mg/d. Overall, one patient (6%) achieved CR lasting for 36 months, and two patients had a transient reduction in marrow blasts from 8% and 7% to less than 5% in both cases. There was no correlation between reduction of angiogenesis markers' levels and response. In a phase I/II trial by Steins et al. [92] a dose-escalating trial was performed to study the safety and efficacy of thalidomide in 20 AML patients. Thirteen patients were assessable for both toxicity and response, tolerating a maximum dose of 200-400 mg/d for at least 1 month. Overall, adverse events were fatigue, constipation, rash, and neuropathy (grade 1 to 2 in most patients). In 4 patients, a partial response, defined as reduction of at least 50% of the blast cell infiltration in the bone marrow accompanied by increases of platelet counts and hemoglobin values, was observed. In parallel, MVD significantly decreased in these 5 patients during treatment with thalidomide. A combination therapy of thalidomide and 5-azacytidine, a hypomethylating drug, was examined in 40 patients with MDS and AML [93]. A hematological improvement was observed in 15 of 36 patients (42%), stable disease was observed in 5 of 36 patients (14%), and 10 of 36 patients (28%) had disease progression. Six patients had CR.

In a phase II study with 44 PMF patients thalidomide was examined as monotherapy [94]. Seventeen of 41 evaluable patients (41%) receiving treatment for at least 15 days showed a response. A complette remission (without reversal of bone marrow fibrosis) was achieved in 4 patients (10%), a partial response was achieved in 4 patients (10%), and hematological improvements of anemia, thrombopenia, and/or splenomegaly were observed in 9 patients (21%).

Lenalidomide, a synthetic compound derived by modifying the chemical structure of thalidomide, has also immunomodulatory and anti-angiogenic properties, while showing lower adverse effects rates [95]. In patients with previously treated relapsed/refractory MM, the combination of lenalidomide with dexamethasone increased the response rate from 22.5% to 59.2% compared to dexamethasone alone [96,97]. In 2 phase III trials lenalidomide in combination with dexamethasone, it showed remarkable response rates and better toxicity profile than thalidomide [96,97]. Lenalidomide was approved in combination with dexamethasone for the second-line treatment of MM.

In phase II studies with lenalidomide monotherapy in patients with symptomatic PMF, the overall response rates were 22% for anemia, 33% for splenomegaly, and 50% for thrombocytopenia [98]. In a combination study of lenalidomide with prednisone, 40 patients with PMF were included [99]. Responses were recorded in 12 patients (30%) and are ongoing in 10 (25%). The median time to response was 12 weeks. Three patients (7.5%) had partial response and nine patients (22.5%) had clinical improvement durable for a median of 18 months. Overall response rates were 30% for anemia and 42% for splenomegaly. Interestingly, all eight *JAK2-V617F*-positive responders experienced a reduction of the baseline mutant allele burden as well.

#### Proteasome inhibitors

Bortezomib (Velcade^®^), a boronic acid dipeptide, is a selective, but reversible proteasome inhibitor [100]. It has been approved for clinical use in humans, in particular for treatment MM and mantle cell lymphoma. Beside its direct anti-tumor effects, anti-angiogenic actions of bortezomib have recently been described in vitro and in vivo [100]. In a study by Roccaro et al. [101] the effect of bortezomib on the angiogenic phenotype of MM patient-derived endothelial cells was examined. Bortezomib inhibited the proliferation of endothelial cells and angiogeneis in a dose-dependent manner.

In a phase III study, patients with MM progressing after at least one prior therapy, were randomized to receive single-agent bortezomib or high-dose dexamethasone [102]. Alltogether 669 patients were included. Time to progression was significantly prolonged in the bortezomib treatment arm (median, 6.2 months) compared with the dexamethasone arm (median, 3.5 months). Analysis of overall survival done on the interim database (with 20% of events) showed the superiority of bortezomib for patients. The response rate (complete plus partial response) with bortezomib was also superior to dexamethasone (38% versus 18%). Adverse events on the bortezomib arm were similar to those previously observed in phase II studies; some notable adverse events being asthenia, peripheral neuropathy, thrombocytopenia, and neutropenia. In another phase III study, bortezomib, melphalan, and prednisone (VMP) was examined versus melphalan and prednisone (MP) in previously untreated symptomatic MM patients ineligible for high-dose therapy [103]. VMP resulted in a 35% reduced risk of death compared to MP and prolonged overalls survival. In a phase I/II study by Richardson et al. [104] the combination lenalidomide, bortezomib, and dexamethasone was evaluated in front-line myeloma. The partial response rate was 100% in both the phase II population and overall, with 74% and 67% each achieving very good partial response or better. The combination lenalidomide, bortezomib, and dexamethasone demonstrated favorable tolerability and was highly effective in the treatment of newly diagnosed myeloma. In a phase I study, bortezomib was added to induction chemotherapy in patients with AML [105]. The combination of bortezomib, idarubicin, and cytarabine showed a good safety profile. The recommended dose of bortezomib for phase II studies with idarubicin and cytarabine was 1.5 mg/m^2^. Overall, 19 patients (61%) achieved complete remission (CR) and three had CR with incomplete platelet recovery.

## Conclusions and future directions

Angiogenic and especially VEGF/VEGFR pathways are involved in the pathophysiology of hematological malignancies including multiple myeloma, acute and chronic leukemias, MPN and lymphomas. Although VEGF/VEGFR-related pathways seems to be the most relevant regulators of neoangiogenesis, vasculogenesis and recruitment of endothelial progenitor cells in such instances, but other pathways are important too. Further, VEGF/VEGFR interactions can stimulate proliferation, migration and survival of leukemia/lymphoma cells by autocrinous and paracrinous loops. Novel agents, targeting VEGF, its receptors, and other angiogenic pathways, are in various stages of clinical development and investigation in hematological malignancies. As we know from the the treatment of solid tumors, combination therapies of different anti-angiogenic molecules with chemotherapy or irradiation increases treatment efficacy. Especially, as blocking VEGF activity has been shown to sensitize the vasculature and improve the delivery of cytotoxic drugs to tumor and endothelial cells. However, not all patients treated with anti-angiogenic therapies benefit from this kind of therapy and in most cases, the effect is transient. Therefore, there is an urgent need for biomarkers to identify patients likely to benefit from anti-angiogenic treatments, to select the optimal dose to minimize side effects, and to understand the mechanisms of resistance. Preclinical models suggest multiple mechanisms involved in acquired or primary resistance against anti-angiogenic therapies. Finally, also these "targeted therapies" has side effects profiles which must be considered carefully.

## Competing interests

The authors declare that they have no competing interests.

## Authors' contributions

MM and KM selected publications for the review, drafted manuscript. Both authors read and approved the final manuscript.
